# Single-stranded heteroduplex intermediates in λ Red homologous recombination

**DOI:** 10.1186/1471-2199-11-54

**Published:** 2010-07-29

**Authors:** Marcello Maresca, Axel Erler, Jun Fu, Anne Friedrich, Youming Zhang, A Francis Stewart

**Affiliations:** 1 Genomics, Technische Universität Dresden, BioInnovationsZentrum, Tatzberg 47-51, D-01307 Dresden, Germany; 2 Gene Bridges GmbH, BioInnovationsZentrum, Tatzberg 47-51, D-01307 Dresden, Germany

## Abstract

**Background:**

The Red proteins of lambda phage mediate probably the simplest and most efficient homologous recombination reactions yet described. However the mechanism of dsDNA recombination remains undefined.

**Results:**

Here we show that the Red proteins can act via full length single stranded intermediates to establish single stranded heteroduplexes at the replication fork. We created asymmetrically digestible dsDNA substrates by exploiting the fact that Redα exonuclease activity requires a 5' phosphorylated end, or is blocked by phosphothioates. Using these substrates, we found that the most efficient configuration for dsDNA recombination occurred when the strand that can prime Okazaki-like synthesis contained both homology regions on the same ssDNA molecule. Furthermore, we show that Red recombination requires replication of the target molecule.

**Conclusions:**

Hence we propose a new model for dsDNA recombination, termed 'beta' recombination, based on the formation of ssDNA heteroduplexes at the replication fork. Implications of the model were tested using (i) an *in situ *assay for recombination, which showed that recombination generated mixed wild type and recombinant colonies; and (ii) the predicted asymmetries of the homology arms, which showed that recombination is more sensitive to non-homologies attached to 5' than 3' ends. Whereas beta recombination can generate deletions in target BACs of at least 50 kb at about the same efficiency as small deletions, the converse event of insertion is very sensitive to increasing size. Insertions up to 3 kb are most efficiently achieved using beta recombination, however at greater sizes, an alternative Red-mediated mechanism(s) appears to be equally efficient. These findings define a new intermediate in homologous recombination, which also has practical implications for recombineering with the Red proteins.

## Background

Homologous recombination (HR) is central to all replicating cells because it is a main pathway for the rescue of replication fork catastrophes [1,2]. When a replication fork encounters a single-strand nick or unrepaired DNA damage, a double-strand break is generated and the replication fork collapses. HR serves to reset the replication fork through a process involving a 5' to 3' exonuclease or helicase, which generates a 3'-ended single-stranded region from the double-strand break. Single-strand binding proteins then promote joint molecule formation by hybridization between the single-stranded region and its recently replicated complementary sequence. Once the complementary sequence is found, the replication fork can be restarted.

Single-strand binding proteins involved in HR promote the formation of joint molecules in two different ways, either strand invasion or annealing. Strand invasion requires a RecA/RAD51 superfamily member to mediate the search for complementarity between the single-stranded DNA (ssDNA) region and double-stranded DNA (dsDNA) through a triple-stranded intermediate termed a displacement loop (D-loop). Annealing is mediated by single-strand annealing proteins (SSAPs) between complementary ssDNAs [3]. By sequence alignment, three families of SSAPs have been defined based on RAD52, RecT/Redβ and Erf [4]. Recently we discovered that the RAD52 and RecT/Redβ families are related [5], suggesting that annealing mechanisms related to HR may share a common basis.

HR is also central to the technologies of gene targeting and recombineering. Gene targeting is the process whereby the genome of a living cell is altered by the introduction of recombinant DNA into a specific site, mediated by the endogenous HR machinery [6-9]. Recombineering mainly refers to the engineering of recombinant DNA by HR [7,10-12]. Both gene targeting and recombineering usually involve the generation of a linear dsDNA copy of a segment of the chosen target DNA. A stretch of heterologous dsDNA, which usually contains a selectable gene, is introduced into the middle of the copy. The incorporation of the linear dsDNA by HR incorporates the central heterologous sequence and so alters the target by 'replacement'. This application of HR differs from endogenous HR because it does not involve the repair of a DSB, rather it is stimulated by the two DSB-like ends of the linear copy.

**Figure 1:**
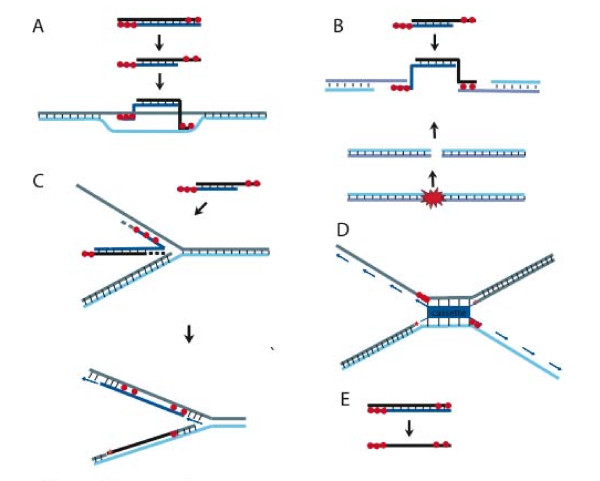
**Proposed recombination models based on a linear dsDNA substrate**. (A) Strand invasion model. A linear DNA is resected on both ends by a 5' to 3' exonuclease (e.g. Redα, RecE, not shown) and the single stranded 3' tails are bound by a recombinase (Redβ or RecT; red circles) to generate nucleoprotein filaments on each end of the linear DNA, which promote a double-crossover joint molecule by strand invasion into target dsDNA. (B) DNA annealing model. Exonucleolytic resection and recombinase assembly is the same as A. However, the nucleoprotein filaments anneal directly to complementary single stranded 3' tails generated from a DSB in the target DNA molecule. (C) Chicken foot model. Exonucleolytic resection and recombinase assembly is the same as A. One end of the linear DNA anneals to a single-stranded region at the lagging strand of the replication fork. A 'chicken foot' intermediate is formed. Backtracking of the chicken foot intermediate by branch migration reestablishes the replication fork (adapted from [7]). D) Bridge model. The linear DNA is inserted in between two colliding replication forks. Each nucleoprotein filament at the ends of the cassette (shown in blue) anneals to the lagging strand of one or the other replication fork thereby inserting the cassette in the middle of the two replication forks (adapted from [7]). E) Schematic illustration of an alternative digestion product after 5' to 3' Redα exonuclease activity. Instead of degradation from both ends of the linear dsDNA, Redα starts only at one end and complete digestion leaves an intact single-strand behind.

The standard model for replacement targeting by HR involves 5' to 3' resection at both ends (Figure 1A), followed by a double crossover [13-16]. However some evidence indicates that other explanations may be valid. Two alternatives to the standard model were discussed by Paques and Haber based on observations made in *S. cerevisiae *[15]. They favoured the evidence of Morrow *et al *[17] for a double primed replication event that has similarities to break induced replication (BIR), leading to extensive chromosomal duplication. They also mentioned the data of Leung *et al *[18] who proposed a model based on the assimilation of a solo single-stranded intermediate.

Here we focus on recombination mediated by the Red proteins of λ phage. The Red operon includes a 5' to 3' exonuclease, Redα [19-21], an SSAP, Redβ [5,22,23] and Redγ, which is an inhibitor of the major *E. coli *exonuclease, RecBCD [24,25]. Whereas the role for Redα to generate ssDNA regions from dsDNA ends is clear, questions regarding the ability of Redβ to mediate strand invasion have been raised [3,26,27]. Kuzminov [3,28] argued convincingly that Redβ can only mediate annealing. However the functional discovery of apparent Redβ strand invasion activity in the absence of RecA [29,30] presented a conundrum. Ellis *et al *[31] provided part of the solution. They observed a strand specific bias in site-directed mutagenesis mediated by Redβ. Single-stranded oligonucleotides that could serve as primers for Okazaki fragment synthesis were more efficient than their complementary oligonucleotides. Hence the model was proposed that Redβ anneals oligonucleotides into single-stranded regions at the replication fork [7,31,32]. This model gained further support from the observation that removal of the mismatch repair pathway enhanced oligonucleotide-directed mutagenesis [33,34].

Whereas this model explains oligonucleotide-directed HR, the mechanism of Red-mediated dsDNA HR remains unexplained. The standard model for replacement HR has been favoured [7,35,36]. However this double crossover model encounters a problem if Redβ cannot catalyze strand invasion. Hence the mechanism illustrated in the diagram of Figure 1A is not possible without modification. An alternative to the standard model invokes the occurrence of an adventitious DSB near the chosen targeting site (Figure 1B). This is possible but the high HR efficiencies achieved using Red recombination cast doubt on this presumption. Accordingly, Court *et al *[7] proposed two further models based on either a chicken foot intermediate (Figure 1C) or the establishment of a bidirectional replication fork (Figure 1D). Neither of these two models is entirely satisfactory. The chicken foot intermediate, as part of the RecG pathway [37], serves to pass damaged nucleotides. However Red recombination can insert thousands of base pairs of heterologous sequence and removal of RecG increases Red recombination efficiencies, indicating that RecG ejects recombination intermediates [38]. The bidirectional replication fork may be reminiscent of BIR, but is without support from evidence. More recently, a template-switching model based on invasion of the replication fork by the 3' end of an ssDNA/dsDNA product of Redα exonuclease digestion has been proposed [39]. However this model cannot explain replacement HR without some modification or extension.

An accidental discovery led us to reconsider the conundrum of Red-mediated dsDNA HR. Inadvertently we noticed a significant number of very late growing colonies produced by a boiled dsDNA control. Examination of these colonies revealed that they all contained the intended recombination (data not shown). Although we later found that boiled dsDNA is very inefficient compared to optimal recombineering practice, this unexpected observation led us to re-evaluate our presumptions and consider a new model for replacement HR by Red (Figure 1E). Rather than beginning with symmetrical resection of the linear substrate by Redα, here we consider the idea that Redα eliminates one strand entirely leaving the other intact. Then Redβ mediates the incorporation of the intact strand into the replication fork to effect replacement HR.

## Results

Most recombination experiments described here employed linear substrates that included 50 nt/bp of continuous sequence identity to the target molecules at each end (termed the 'homology arms'), flanking a gene encoding resistance to an antibiotic. The target molecules were intact circles, being either plasmids, Bacterial Artificial Chromosomes (BACs) or the *E. coli *chromosome. Recombination was scored as colonies that acquired resistance to the corresponding antibiotic under conditions where all antibiotic resistant colonies were due to the intended recombination, as evaluated by DNA analyses (data not shown). The recombination reactions were carried out with our standard protocol, which has been optimized for convenience and productivity. All experiments were performed in *recA *strains.

### Replication is required for Red recombination with dsDNA substrates

**Figure 2:**
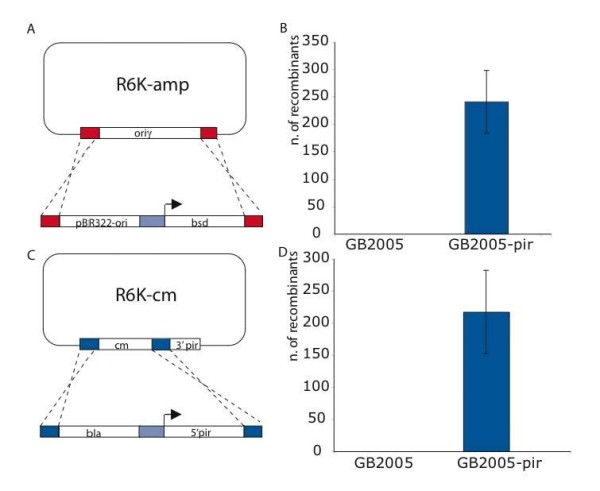
**Replication is required for Red recombination**. (A) Schematic illustration of an experiment to replace the oriγ replication origin in an R6K-amp plasmid with the pBR322 replication origin and the *bsd *gene for blasticidin resistance. (B) Quantification of recombinant colonies from the experiment shown in (A). Recombination only occurred when the *pir *gene was expressed in trans from the *E. coli *chromosome (strain GB2005-pir). No recombination product was found in the absence of *pir *(strain GB2005). (C) Schematic illustration of an experiment to rebuild the *pir *gene in an R6K plasmid using an ampicillin resistance gene *bla *and a 5' fragment of the *pir *gene, which completes the *pir *gene when fused with the 3' end of the *pir *gene. (D) Quantification of recombinant colonies of the experiment depicted in (C). Similar to the result shown in (B), recombination only occurred when the Pi protein was expressed in trans.

In the absence of RecA, dsDNA recombination by the Red proteins requires replication, however the mechanism remains undefined [39]. To define the step at which replication is required for Red recombination with dsDNA substrates, we performed the experiment shown in Figure 2A. We used an R6K plasmid, which requires the Pi protein to replicate [40,41], as well as a linear dsDNA fragment that included a colE1 origin and the blasticidin resistance gene. The homology arms were chosen so that recombination would replace the R6K origin with the colE1 origin. Thereby the recombination product will have a functioning colE1 replication origin. If replication is required to initiate recombination, then the Pi protein must be present so that the R6K plasmid is replicating. On the other hand, if replication is only required to amplify the initiating recombination complex, then the insertion of the colE1 origin should suffice.

The two DNAs were co-electroporated into an *E. coli *strain that contained Redαβγ with or without expression of Pi (strains GB2005 and GB2005-pir respectively). Recombination only occurred in the presence of Pi, demonstrating a need for replication of the R6K plasmid before recombination (Figure 2B). To support this result, we altered the experiment to direct recombination towards the *pir *gene (Figure 2C). Rather than exchange the origin of replication, here recombination will restore expression of Pi, which will permit replication of the plasmid. However no plasmid replication occurred without supplying Pi in trans at the start of the experiment (Figure 2D).

These results show that the target molecule must be replicating to permit the initiation of dsDNA recombination.

### Rethinking the dsDNA intermediate in Red recombination

**Figure 3:**
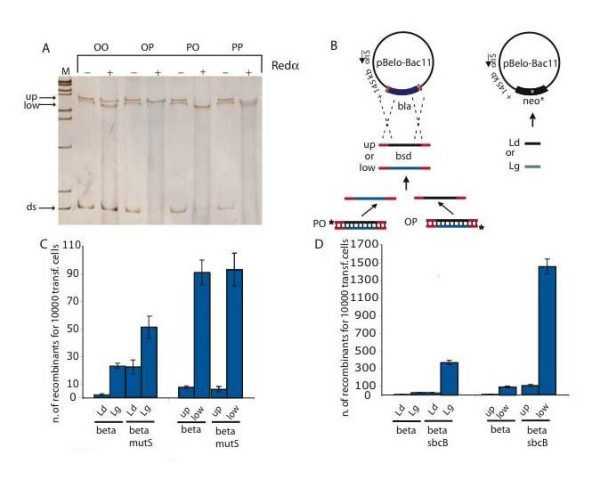
**Recombination and oligonucleotide-mediated mutagenesis using ssDNAs**. (A) SSCP-PAGE gel of phosphorylated (P) and hydroxylated (O) recombination cassettes in all four combinations (OO, OP, PO, PP) with (+) or without (-) Redα *in vitro *digestion. The lower bands show undenatured dsDNA (ds) and the upper bands show the slower migrating secondary structures of the single-strands after heat denaturation (up and low). M - size markers. (B) Schematic representations of the recombination reactions, which either replaced the ampicillin resistance gene (*bla) *in pBelo-BAC11 with the 500 nt ssDNA products shown in A, which contain the blasticidin resistance gene (*bsd*; left side) or repaired a 4 nt mutation in the kanamycin resistance gene (*neo) *using 100 nt single-stranded oligonucleotides (right side). Only Redβ was expressed in these experiments. The oligonucleotides were either complementary to the leading (Ld) or lagging (Lg) strands. (C, D) Quantification of recombinant colonies using the experimental designs shown in (B) and comparison of the wild type (wt) to either *mutS *(C) or *sbcB *(D) strains.

Previous ideas about Red recombination with dsDNA substrates have assumed that the reaction initiates with action by Redα exonuclease to create a symmetrically resected ssDNA/dsDNA intermediate (as illustrated in Figure 1A) that then hybridizes to form some kind of joint molecule. To challenge this assumption and test the possibility that the recombination intermediate is a full-length ssDNA, we exploited the fact that Redα prefers to begin exonuclease activity on a 5' phosphorylated rather than hydroxylated (dephosphorylated) end [21,42]. A dsDNA substrate was prepared carrying either phosphorylated (P) or hydroxylated (O) 5' ends in all four combinations (OO, OP, PO, PP) as illustrated in Figure 3. These substrates were digested with Redα *in vitro *and the products analyzed by single-strand conformation polymorphism (SSCP), which can separate complementary single-strands by gel electrophoresis [43,44]. As can be seen in Figure 3A, Redα digested only the phosphorylated strand in the OP and PO substrates, whereas the OO substrate was not digested on either strand and the PP substrate was digested on both, producing some different ssDNA products.

Subsequently we used these OP and PO ssDNA products in a recombination reaction to insert the blasticidin (*bsd*) resistance gene into a BAC (Figure 3B). Because Redα is not required for Redβ mediated recombination with single-stranded oligonucleotides [31,32,45], the host cells contained only Redβ. In parallel as a positive control, we performed a recombination reaction with single-stranded oligonucleotides to repair a mutation in the kanamycin resistance gene (*neo*) and so restore kanamycin resistance. As expected from previous observations, the oligonucleotide that can serve as a primer for Okazaki-fragment synthesis (Lg) produced more recombinants than the complementary oligonucleotide (Ld; Figure 3C). The same effect was observed with the ssDNA substrates. That is, the ssDNA that can prime Okazaki-fragment synthesis (low) delivered many more recombinants than the complementary strand. Notably, the two assays could be distinguished by mutations in the mismatch repair pathway (*mutS*), indicating the two reactions are not identical. As shown before [33,34], mutations in the mismatch repair pathway enhanced oligonucleotide-directed mutagenesis and the enhancement was greater for the leading strand oligonucleotide (Ld). However, we found no *mutS *effect on ssDNA recombination (Figure 3C). Conversely, mutating the 3' to 5' exonuclease I, *sbcB *[46,47], significantly enhanced both oligonucleotide and ssDNA directed recombination indicating that both assays are similarly sensitive to 3' exposed ssDNA ends.

### Testing the ssDNA intermediate in vivo

**Figure 4:**
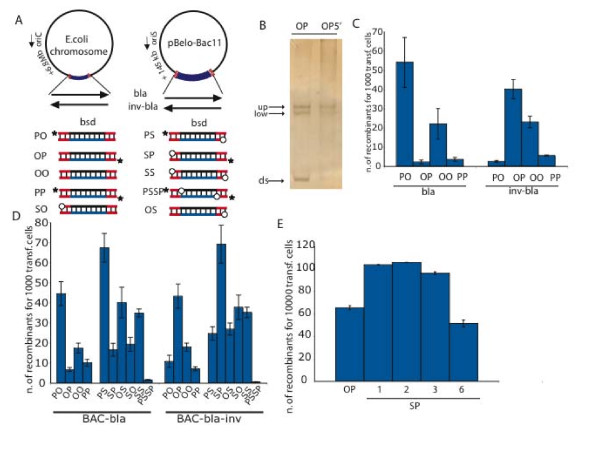
**In vivo evidence for the ssDNA intermediate**. (A) Schematic representations of recombination reactions to replace the ampicillin (*bla*) with the blasticidin (*bsd*) resistance gene in either the *E. coli *chromosome or pBelo-Bac11. The *bla *gene was present in both orientations (*bla, inv-bla*) to alter its orientation to the direction of replication, which is indicated by an arrow beside the origin, either oriC or oriS. The *bsd *gene was PCR amplified to possess different 5' ends as indicated, either phosphorylated (P, black stars), hydroxylated (O, no symbol) or phosphothioated (S, open circles) in various combinations. PSSP indicates that both 5' ends were phosphorylated and the first two backbone linkages were also phosphothioated. All three Red proteins (α, β, γ) were expressed in these experiments. (B) SSCP-PAGE gel of a hydroxylated/phosphorylated linear dsDNA substrate (OP) after transformation *in vivo*. The lower band represents dsDNA (ds) and the upper bands represent the slower migrating secondary structures of the single-strands after heat denaturation (up and low). DNA was harvested from cells immediately after electroporation (OP) or after incubation for 5 minutes at 37°C (OP5'). (C, D) Quantification of recombinant colonies using the experimental design shown in (A). The targeted loci were either located on the *E. coli *chromosome (C) or on the pBelo-Bac11 BAC (D) in direct (*bla*) or inverted (*inv-bla*) orientation. (E) Kanamycin-resistant colonies after repair of pBelo-Bac11-neo* BAC using dsDNA with zero (OP), one (SP-1), two (SP-2), four (SP-4) or six (SP-6) consecutive phosphothioated bonds at one 5' end. The other 5' end was phosphorylated (P).

To determine whether dsDNA recombination can be processed through a full-length ssDNA intermediate *in vivo*, we employed the four linear dsDNA substrates (OO, OP, PO, PP) in a recombination assay in the presence of Redα as well as Redβ (and Redγ). The experimental design is shown in Figure 4A. Recombination was directed to either a BAC or the *E. coli *chromosome. In both cases, the target was established in both orientations (bla and inv-bla), which alters the recombination reaction with respect to the direction of replication. Using an asymmetric substrate (OP), SSCP revealed that Redα rapidly generated the ssDNA intermediate *in vivo *(Figure 4B). Recombination was most efficient with the asymmetrically phosphorylated substrate whose hydroxylated 5' strand could prime lagging strand synthesis, henceforth referred to as the 'LSP' for lagging strand primer. Inverting the target inverted the strand preference, demonstrating that the PO and OP substrates were both proficient. The same conclusions could be drawn from both the BAC and chromosomal assays (Figure 4C, D). Notably, the next best substrate in all configurations was the doubly hydroxylated one (OO) and in this case inverting the target had no effect.

To challenge these findings in an additional way, we made substrates with phosphothioated bonds at the 5' ends. We first established that two consecutive phosphothioate bonds is optimal and superior to hydroxylated ends (Figure 4E). Hence we generated dsDNA substrates with two phosphothioated bonds at the 5' end (S) in combination with phosphorylated (P) or hydroxylated (O) 5' ends. Again, the most efficient SP orientation had the phosphothioate at the 5' end of the LSP (Figure 4D). Consistent with predictions, the asymmetrically phosphothioated/phosphorylated (SP, PS) LSPs were more efficient than the hydroxylated/phosphorylated (OP, PO) or phosphothioated/hydroxylated LSPs. Furthermore permutations of S and O, like the OO substrate, also delivered good recombination efficiencies. The SO substrates displayed a limited strand preference, whereas the SS substrate showed none.

All of these results are consistent with the following conclusions; (i) 5' phosphothioates or 5' end hydroxylation enhanced recombination of the LSP because they convey resistance to 5' - 3' exonuclease activity; (ii) this enhancement is amplified when the 5' end of the other strand is phosphorylated because rapid degradation of the unfavourable strand increases the yield of full-length, single-stranded, LSP; (iii) when both 5' ends are blocked to the exonculease, it is possible that another activity such as a helicase separates the strands so that the full-length LSP can initiate recombination.

**Figure 5:**
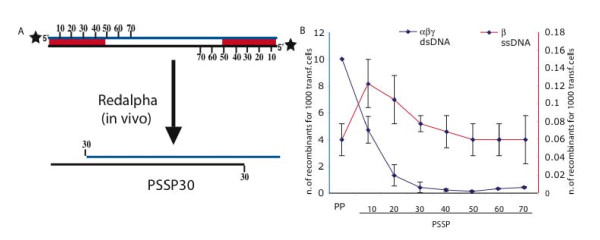
**Red recombination of symmetrically resected dsDNA substrates**. (A) Schematic representation of linear PSSP dsDNA substrates showing the distance (in nt) to two phosphothioate bonds that were symmetrically positioned from each 5' end. Additionally, phosphates were present at both 5'ends (black stars). Below the PSSP30 substrate is illustrated after Redα digestion exposing 30 nt ssDNA tails at each end; (B) Recombination efficiencies of the PSSP substrates. Either Redα, Redβ and Redγ (blue line, left ordinate) or only Redβ (red line, right ordinate) were expressed. In the second case (only Redβ) the dsDNA substrates were heat denatured prior to electroporation.

As a further test, we designed an experiment to evaluate the recombination efficiencies of symmetrically resected dsDNA intermediates. A pair of phosphothioates were symmetrically placed at increasing distances from the 5' ends of a substrate as illustrated in Figure 5. That is, the phosphothioates were either 10, 20, 30, 40, 50, 60 or 70 nt from each 5' end. Both 5' ends were phosphorylated to promote Redα exonuclease activity. We reasoned that Redα would resect both ends until reaching the phosphothioate bonds, thereby generating the symmetrically resected substrates that were previously believed to be the optimal recombination intermediates. By this previous logic, the series of phosphothiolated substrates up to 70 nt was chosen to include the presumptive best intermediates that have both 50 nt homology arms exposed as ssDNA and the region in between as double-stranded. However, these substrates were very inefficient (Figure 5B), particularly when compared to the SS substrate (Figure 4D). To control for the quality of the substrates, they were boiled to separate the strands and used in a recombination assay mediated by Redβ only. Although this Redβ-only recombination reaction is very inefficient, each of the internally phosphothioated substrates were equivalent for recombination after boiling demonstrating their integrity for recombination.

**Figure 6:**
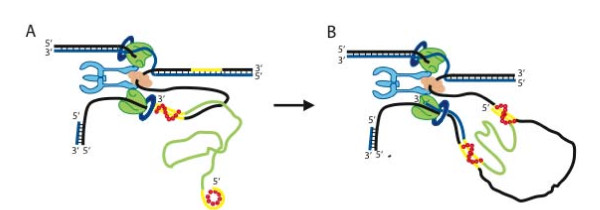
**Model for a solo single-stranded DNA intermediate in Red recombination**. Model for recombination at the replication fork. Annealing of an ssDNA molecule to complementary regions on the lagging strand at the replication fork is depicted. The ssDNA molecule comprises two flanking homology arms (~50 nt; yellow), interspaced by a heterologous sequence (light green). The Redβ annealing intermediate is shown as a curved line of red dots. The leading strand is shown in blue, lagging strand in black, DnaB helicase in light orange, the two Pol III holoenzymes are green, which are tethered to the γ/τ clamp loader (light blue), and the β sliding clamps are dark blue rings. (A) The Redβ-ssDNA protein complex anneals the 3' end first, which then primes DNA synthesis for an Okazaki fragment. (B) After replication fork progression, the second homology region becomes exposed and annealing of the 5' homology arm creates the ssDNA heteroduplex intermediate.

Taken together, this evidence supports a new model for Red replacement HR mediated through a single-strand that forms a heteroduplex at the replication fork, as illustrated in Figure 6. We call this model 'beta' recombination for convenience. To challenge the model, we examined several implications.

### Evidence for a heteroduplex intermediate in Red replacement HR

**Figure 7:**
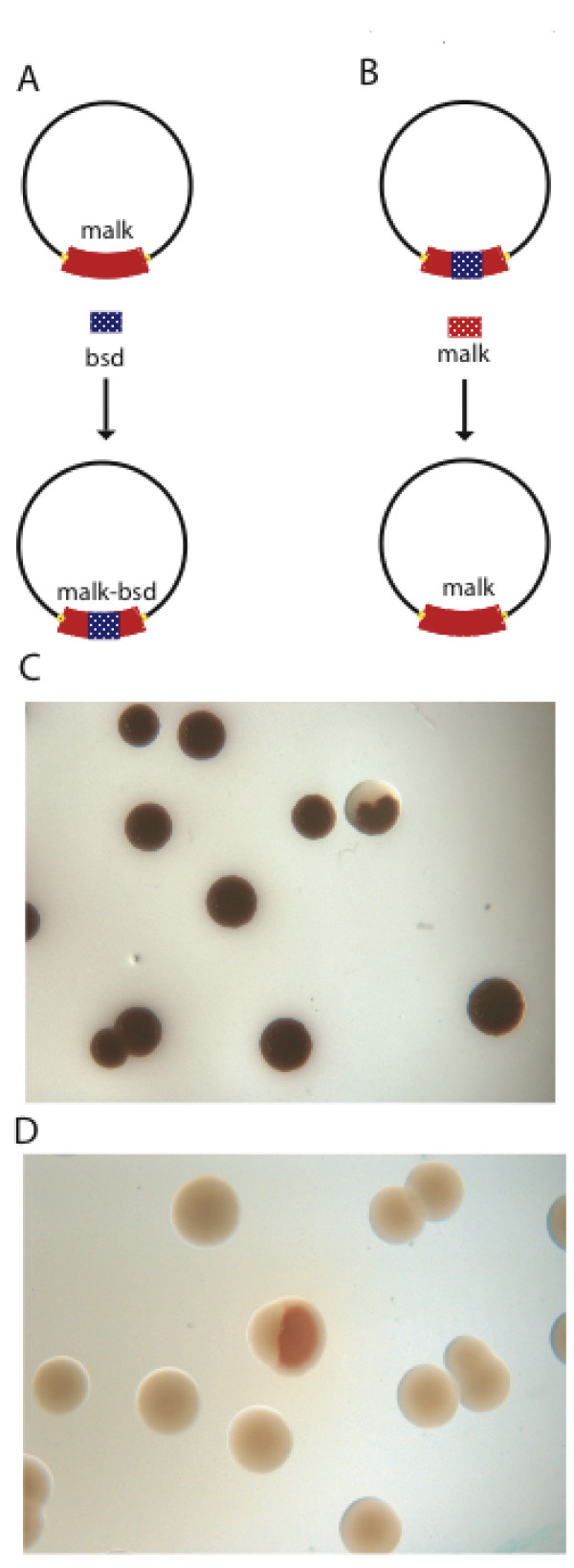
***In situ *recombination assay based on colony colour**. (A) Schematic illustration of the recombination assay, which leads to an insertion of the blasticidin resistance gene (*bsd) *into the genomic *malK *locus. Insertion mutates the *malK *gene, so recombinant colonies are white when plated on MacConkey agar. (B) Schematic representation of the reverse recombination event, which restores the functionality of the *malk *gene by deleting *bsd*, which was inserted in (A). The linear substrate was a 400 bp dsDNA fragment of the *malK *gene. (C) Picture of red (non-recombinant) and half white/red (recombinant) colonies obtained using the design shown in (A). (D) Picture of white (non-recombinant) and half red/white (recombinant) colonies obtained using the recombination design shown in (B).

The beta recombination model predicts that the recombination intermediate is initially a heteroduplex on the lagging strand of both mutant and parental strands. If this heteroduplex is replicated, the product should be one daughter that is parental and one that is mutant. To look for this possibility, we developed an *in situ *recombination assay based on colony colour. On MacConkey agar, cells carrying an intact *malK *gene give a red colour whereas mutants are white [48]. The assay was performed in two directions, one to mutate the *malK *gene and the other to restore it. After brief incubation to permit recombination, cells were plated out at single cell dilution and cultured until the colony colour was apparent (Figure 7). Because an *E. coli *cell can harbour two or more copies of any gene (after it has been replicated, before cell division), we performed these experiments as a time course of brief incubation before plating. As presented in Table 1, almost all recombination events at the earliest time point resulted in two-colour colonies, indicating that recombination produced a heteroduplex as predicted. Upon longer incubation times, the proportion of single colour colonies increased, which indicated an increasing proportion of colonies formed by cell division before plating as expected.

**Table 1:** Time course of recombination products at *malK *as scored by colony colour

Time (min) before plating	Half white/red colonies		Full white no./7000
	Percent	no./7000	
5	93	13	1

10	92	11	1

15	86	12	2

20	81	13	3

25	76	13	4

30	77	10	3

60	40	6	9

### Asymmetry of the homology arms

The beta recombination model implies that the homology arms are not equivalent. That is, the homology arm at the 3' end of the LSP should anneal first, followed by annealing of the 5' LSP homology arm. Also, the homology arms on the other strand should be unimportant. These asymmetries permit certain tests.

**Figure 8:**
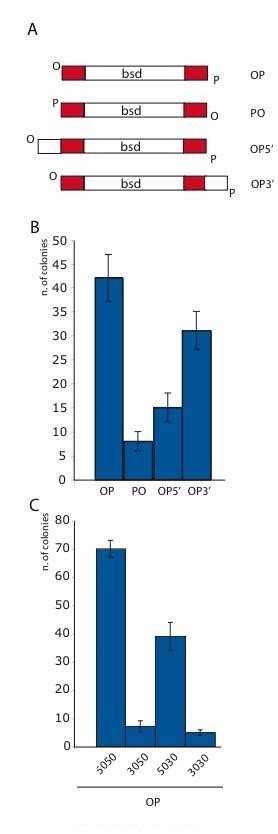
**Terminal non-homologies indicate an underlying asymmetry**. (A) The substrates are illustrated showing the location of the 50 bp homology arms (red), hydroxylated (O) or phosphorylated (P) 5' ends and the additional non-homology (30 bp) on one end or the other. OP5' refers to the addition of the non-homology onto the 5' end of the LSP. OP3' refers to the addition of the non-homology on the 3' end of the LSP. (B) Results using the substrates shown in (A) after recombination into a BAC in the presence of Redαβγ. (C) The same assay as in (B) except that the homology arms of the PO substrate (50 50) were altered to be shorter on the 5' end of the LSP (30 50), the 3' end (50 30) or both (30 30).

First, we tested the impact of adding non-homologous sequence to the 3' or 5' ends of the LSP (Figure 8A). When the non-homology was present on the 5' end of the LSP (OP5'), recombination was impaired more than when it was on the 3' end (OP3'). This suggests that additional non-homologous sequences on the 3' end, which will impede priming for Okazaki fragment DNA synthesis, are easily removed. Whereas additional non-homologous sequences at the 5' end are a more substantial impediment, possibly because they interfere with the ligation step required to complete the mutant strand.

Second we tested the impact of altering the length of the 3' or 5' homology arms of the LSP by making one homology arm shorter (30 rather than 50 nts; Figure 8B). As expected, recombination was most efficient when both homology arms were 50 nts (50 50). Reducing the 5' end (30 50) dramatically reduced efficiency whereas reducing the 3' end (50 30) had a moderate effect. Notably reduction of both homology arms (30 30) was similar to the 5' end reduction only, indicating that the length of the 5' arm is the critical element.

These tests lend support to the beta recombination model by illustrating the non-equivalence of the two homology arms.

### The length of deletion has little effect but the length of insertion is critical

**Figure 9:**
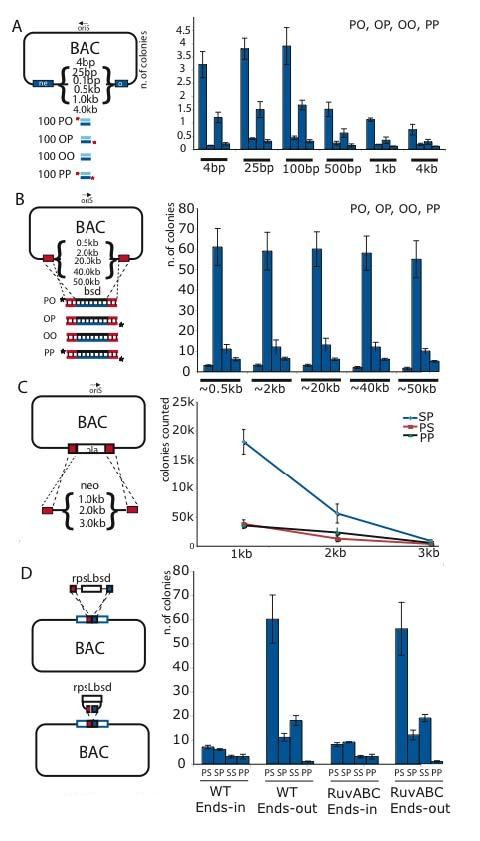
**Increasing the deletion size has little effect but increasing the substrate size indicates limits to beta recombination**. (A) Schematic illustration of an assay using a BAC carrying variously sized insertions in the *neo *gene, from 4 to 4000 bps as indicated, which were deleted by a 100 bp fragment to restore kanamycin resistance. Results are plotted at the right. (B) Schematic illustration of an assay using a BAC assay with one homology arm (red) placed at various distances from 0.5 to 50 kb to the other homology arm (red) as indicated. The region between these two sequences was deleted by insertion of a 500 bp fragment carrying blasticidin resistance as plotted to the right. (C) Schematic illustration of an assay to insert either 1, 2 or 3 kb fragments carrying the *neo *gene into the same place on a BAC. The fragments were synthesized to have 5' phosphorylated (P) or phosphothioated (S) ends as indicated in the plotted curves to the right. (D) Schematic illustration of an assay showing the insertion of a 1kb fragment carrying the *rpsL *and blasticidin (*bsd*) genes into the same site on a BAC. The 1kb fragment was identical except that the homology arms were arranged for ends-out (replacement, above) or ends-in (below) recombination, and synthesized with combinations of 5' phoshorylated (P) or phosphothioated (S) ends as indicated in the plot to the right. The experiments were performed in our standard host (GB2005 = WT) or the same host after deletion of *ruvabc*.

The experiments performed above used small cassettes (< 1 kb) for replacement targeting to achieve similarly sized deletions (<1 kb) in the target molecule. To determine whether larger deletions or insertions influence the mechanism, we built assays for the experiments of Figure 9. In particular we were interested to look at deletions across size ranges including 1 kb, which is the average size of Okazaki fragments in *E. coli*, in case a discontinuity around 1 kb could be observed. For Figure 9A, we made a series of substrates by inserting from 4 to 4000 bps into the kanamycin resistance gene (*neo*) in a BAC. These insertions were then deleted by a short (100 bp) fragment to restore the *neo *gene. The 100 bp fragment was generated in the four variations of 5' phosphorylation and hydroxylation as illustrated. In a complementary assay to examine larger regions, one homology region was first inserted at various distances from the other around a BAC from 0.5 to 50 kb (Figure 9B). Then the region in between was deleted by replacement with the blasticidin gene using a 500 bp fragment that was differentially 5' phosphorylated. Although the shorter deletions (< 500 bps) were most efficient, there was overall very little drop in efficiency with increasing size of deletion from 0.5 to 50 kb. Furthermore, the strand preference for the LSP was maintained throughout all deletion sizes, even to 50 kb.

In contrast to deletions, varying the insertion size revealed a vital insight. The efficiency of beta recombination, that is the preference for the LSP over its complementary strand, decreased with increasing size (Figure 9D). At 1 kb, the LSP preference was large, whereas at 2 kb it was diminished and at 3 kb there appeared to be no preference for the LSP over the other ways to establish recombinants, including via the PP substrate, which is presumably symmetrically resected by Redα. This relationship between length and efficiency of beta recombination could be due to limitations in the processivity or speed of Redα exonuclease activity [21,42]. Whatever the reason, this result not only indicates an important practical relationship between insertion size and beta recombination efficiency but also the possibility that dsDNA recombination mediated by the Red proteins can occur by another mechanism in addition to beta recombination.

### Ends-in recombination illustrates a second pathway for Red recombination

To establish whether Red dsDNA recombination can operate through another mechanism(s), we compared replacement recombination, also known as 'ends-out', with 'ends-in' recombination using the same target and homology regions (Figure 9D). As described above, replacement recombination showed the expected preference for the LSP. However insertional recombination did not show a strand preference. Notably removal of RuvABC had no impact on either beta recombination or the alternative pathway. Furthermore the beta recombination pathway is clearly more efficient than the alternatives at insert sizes of less than 3 kb.

## Discussion

We present a new model for replacement targeting by the lambda Red pathway based on a heteroduplex intermediate that incorporates only one strand of the dsDNA substrate, which we term 'beta' recombination (Figure 6). In contrast to a double crossover model, beta recombination does not involve symmetrical resection of the dsDNA substrate from each end by the 5' to 3' Redα exonuclease. Instead one strand is removed completely whilst the other strand remains unresected and contains both homology arms. Like oligonucleotide-directed mutagenesis mediated by Redβ, the strand that delivers more recombinants is the strand whose 3' end can serve as a primer for lagging strand synthesis (termed the LSP). Hence we suggest that this mode of dsDNA recombination, like ssDNA recombination, also occurs at the replication fork.

The reliance of Red recombination on replication was identified a long time ago [49]. However this relationship was subsequently attributed to the replication-dependent generation of recombinogenic dsDNA ends [50] rather than a direct involvement of the replication fork. Furthermore, support for a replication-independent, annealing mechanism was found [28]. Also, the relationship between Red recombination and replication has been complicated by the influence of RecA [36,51-53] presumably because RecA can assist Red recombination by mediating replication-independent strand invasion. In the presence of replication, RecA can make a substantial contribution to Red mediated recombination under certain circumstances [51] but not others [32,35,54,55]. The discovery, that the Red proteins and the closely related RecE/RecT proteins can mediate an apparent strand invasion activity in the absence of RecA [29,30] led to a new linkage between Red recombination and replication. Because Red-mediated oligonucleotide-directed mutagenesis showed strand preference according to the direction of replication, annealing of ssDNA primers at the replication fork for lagging strand synthesis was proposed [7,31,32]. Using an *in vivo *assay based on phage infection and restriction cleavage, it has been recently shown that Red-mediated dsDNA recombination requires replication [39]. Here we also show that replication of the target molecule is required for Red-mediated dsDNA recombination (Figure 2).

The beta recombination model (Figure 6) implies that the two homology arms of the dsDNA substrate are not equivalent. The homology arm at the 3' end of the LSP is more likely to prime recombination than the other 3' homology arm. Also, the homology arm at the 3' end of the LSP should anneal before the homology arm at the 5' end of the LSP because its' complementary sequence is exposed earlier at the replication fork. Hence Redβ likely stabilizes annealing of the 3' homology arm while the replication fork continues. Later, when the region complementary to the 5' homology arm is exposed, the 5' homology arm anneals and the heteroduplex that promotes recombination is established. On this point, it was recently shown that the replication fork continues even though cleavage has occurred in the recently replicated DNA [56]. Therefore it appears that the progress of the replication fork in *E. coli *is not sensitive to events behind it. Support for the idea that the two homology arms in a dsDNA substrate are not equivalent for Red recombination has been recently published [57]. These authors showed recombination asymmetries that can be explained by the beta recombination model. Here we show that heterologous sequence at the 3' end of the LSP has little deleterious impact, presumably because it is rapidly cut away in the process of priming replication, whereas heterologous sequence at the 5' end of the LSP interferes with recombination. Also we suggest that the 3' annealed end is rapidly stabilized by priming DNA synthesis, which is supported by observation that the length of the 5' homology arm is more important than the length of the 3' homology arm (Figure 8). These asymmetric effects further support the beta recombination model and have practical implications for recombineering efficiency.

To test the idea that beta recombination involves the proposed ssDNA heteroduplex, we developed an *in situ *assay to see if replication of the heteroduplex generated one wild type and one recombinant daughter, which should be visible as colony sectoring (Figure 7). Colony sectoring was observed however this result could also be generated if the host cell harboured two copies of the target *malK *locus. Therefore we looked at colony sectoring as a time course of recombination before plating. At the shortest time points, virtually all recombinant colonies were two-coloured, whereas increasing the incubation time resulted in a greater proportion of one-coloured recombinant colonies indicating that cell division occurred after recombination and before plating. It also indicates that the majority of recombinant cells carried only one copy of the *malk *locus at the moment of recombination. Taken together with the evidence that Red recombination requires replication of the target, the colony sectoring results support the existence of a heteroduplex intermediate.

We, and others, have previously reported that large deletions can be readily achieved with Red recombination at efficiencies similar to small deletions. However this parameter has not been examined systematically. Here we looked carefully at deletions around the size of an Okazaki fragment (~1 kb) in two different assays but did not see any discontinuity or any substantial loss of efficiency up to deletions of 50 kb. Taken together with evidence supporting the subsequent replication of the heteroduplex after establishment (Figure 7), it appears that the Redβ annealed complex is highly stable *in vivo*. This concords with the high stability of the complex *in vitro *[5,22,23]. Alternatively, a transient Redβ annealed complex could be rapidly stabilized once it has served to prime replication.

Although we present evidence that Redβ recombination with dsDNA is mediated via solo ssDNA intermediates, our evidence does not exclude additional mechanisms. Indeed, we found circumstances where less efficient levels of recombination appear to be evidence of another mechanism (Figure 9), which could be explained by a template-switching model [39]. The Red proteins can also mediate recombination by a simple annealing mechanism [28]. Furthermore the apparent recombination on the leading strand (that is, from the strand complementary to the LSP) requires an explanation that could also be mechanistically quite different. Consequently it seems that the Red proteins, and Redβ in particular, have the capacity to facilitate more than one recombination pathway. Notably, the role of the Red proteins in the lambda phage lifecycle remains unresolved [12,39], although an evolutionary role has been proposed [58]. Our evidence further strengthens the suggestion that the Red proteins promote the switch from theta to rolling-circle replication in the lambda lifecycle, possibly by more than one mechanism.

The beta recombination model also has some practical implications. As with oligonucleotide-directed mutagenesis, knowledge of the direction of replication can be used to optimize recombineering efficiencies. For many applications, the additional efficiency achieved is not necessary, particularly because the OO, SS and even PP configurations usually deliver more than enough product for DNA engineering exercises. However in certain cases, such as high throughput recombineering [59,60], the further gain of efficiency can deliver useful reliability or help achieve complex tasks (unpublished observations). Furthermore we suggest that the beta model may have relevance for applied HR in other systems. Because strand bias in oligonucleotide-directed mutagenesis has also been observed in yeast [61], it will be interesting to test whether asymmetrical dsDNA substrates show a similar efficiency profile. Exploring these parameters in mammalian cells could also be fruitful.

Several other practical points emerged; (i) two consecutive phosphothioates increased recombination efficiency, presumably because they stabilized the 5' end against exonucleases whilst being permissive for the further steps of recombination. Notably the dsDNA substrates with phosphothioates at both 5' ends were the second-most efficient configuration in our experiments. We suggest that these substrates are also utilized by beta recombination, however are made single-stranded by a helicase activity; (ii) identical DNA fragments produced by PCR or restriction digestion usually differ with respect to phosphorylation at their 5' ends. This can lead to different recombination efficiencies; (iii) heterologous extensions beyond the homology arms are better tolerated at 3' but not 5' LSP ends; (iv) shorter cassettes are significantly more efficiently incorporated than longer, which has implications for high throughput and complex recombineering tasks.

## Conclusions

We present evidence supporting a model for homologous recombination mediated by Redβ acting via a single stranded heteroduplex intermediate formed at the replication fork. Although other mechanisms are not excluded, it appears that the remarkable efficiency of Red recombination utilizes this single stranded intermediate. The model has several practical implications for recombineering when highly efficient applications are required.

## Methods

### Strains

All the strains and substrates used in this work were generated by recombineering. GB2005 is a derivative of HS996. The Rac prophage *recE *and *recT*, and DLP12 prophage *ybcC *that encodes a putative exonuclease similar to the Redα, were deleted to generate GB2005. Both deletions were done by insertion of a selectable marker (*kan*) flanked by lox66 and lox71 sites; subsequently the selectable marker was removed by Cre recombination. The *mutS *and *sbcB *deletion strains were generated in a similar way by removing the whole open reading frame of the respective genes, however the cassette used for deletion was the puromycin resistant gene (*puro*) flanked by rox sites. The puro cassette was subsequently excised by Dre recombination [62]. GB2005-pir was obtained by inserting a cassette containing the *pir *gene plus a selectable marker (*kan*) flanked by lox71 and lox66 sites in the *ybcC *locus. And then the selectable marker was removed by Cre recombination.

### Recombineering

All recombination reactions were performed as follows unless stated. Cells carrying a pSC101 vector, either BAD-Redαβγ or BAD-Redβ were passaged by inoculation of 0.5 ml from a fresh overnight culture into 25 ml LB, 5 μg/ml tetracycline and grown at 30°C for 2 hours. Then protein expression was induced by addition of L-arabinose to 0.2% and grown at 37°C for 45 minutes followed by centrifugation at 0°C, resuspension in ice cold 10% glycerol and transfer to an ice cold Eppendorf tube. The cells were then washed 2 times with ice-cold 1 ml of 10% glycerol in the Eppendorf tube and then diluted to 600 μl ice cold 10% glycerol. 30 μl were used for each electroporation in pre-chilled 1 mm Eppendorf 100 μl cuvettes using an Eppendorf 2510 set at 1350 V. After electroporation, the cells were resuspended in L-broth and incubated at 37°C for 45 minutes before plating. All experiments were repeated three times using 50 ng of co-electroporated plasmid as an internal control for electroporation efficiency.

### Oligonucleotide sequences

The sequences of the oligonucleotides used for recombineering can be found in Additional file 1.

### Replication assays

The replication origin in the R6K-amp plasmid was exchanged using a PCR product containing the blasticidin resistance gene (bsd) and the pBR322 origin of replication. A plasmid was built to serve as template for the PCR reaction and homology arms to the R6K plasmid were added by the PCR reaction. Then, co-electroporation of 200 ng of the R6K-amp plasmid DNA with 200 ng of PCR product into cells expressing Redαβγ under arabinose induction in presence (GB2005-pir) or absence (GB2005) of the Pir protein was followed by plating onto blasticidin (50 μg/ml) plates. The restoration of a functional pir gene in a R6Kγ ori plasmid was done by recombining a cassette containing the ampicillin resistance gene (*amp*) plus the 3' end of the *pir *gene into a R6K-cm-pir* plasmid that carried only the 5' end of the pir gene. The PCR template was pR6K-amp-pir*. Both pR6K-cm-pir* and pR6K-amp-pir* were constructed from pR6K-hyg-pir. The recombinants were identified and counted on ampicillin plates (100 μg/ml).

### λ exonuclease (Redα) digestion

was performed to isolate ssDNA from asymmetrically phosphorylated dsDNA. 100 pmol of dsDNA were digested with 1 unit of λ exonuclease (New England Biolabs) for 1 hour at 37°C, followed by purification using gel extraction kit (QIAGEN, Germany). 2.5 volumes of denaturating buffer (95% formamide, 10 mM NaOH, 0.05% xylene cyanol, 0.05% bromophenol blue) was added to 1 volume of digestion product before boiling for 5 minutes at 95°C and placing on ice. 20 μl were loaded per each lane containing 10 μl from the boiled sample and 10 μl from the unboiled sample. Native polyacrylamide gel electrophoresis was used for SSCP overnight (14 hours) at 50 V. ssDNA recombination was performed by electroporating 2 pmoles of ssDNA generated from the *in vitro *Redα digestion. For the ssOR experiment 1 pmole HPLC-purified oligonucletide was used.

The samples for the *in vivo *digestion experiment were prepared using 5 vials of competent cells (each 40 μl), which were each transformed with 170 ng of asymmetrically phopshorylated bsd cassette. 1 ml of L-broth was added to the cells and they were incubated 5 minutes at 37°C. After extracting the DNA using a standard plasmid mini-prep, the 5 DNAs were pooled and loaded as above on an SSCP gel.

### Differentially phosphorylated cassettes

were generated by PCR using differentially end-modified oligonucleotides. Four different kinds of modification were exploited: normal unmodified 5' ends (O oligonucleotides), phosphorylated 5' ends (P oligonucleotides), insertion of 2 phosphothioate linkages between the first and the second and the second and the third nucleotides starting from 5' end (S oligonucleotides) and both phosphorylated 5' ends combined with 2 phosphothioate linkages (PS or SP oligonucleotides).

### Recombinations with differentially 5' end phosphorylated dsDNA

were done by electroporating 2 pmoles and plating 50 μl of the 1 ml resuspension. The pBelo-BAC-bla and pBelo-BAC-inv-bla were generated by inserting the ampicillin resistance gene (*amp*) into the *neo *gene of pBelo-BAC11-neo* in both orientations. The same cassette was also inserted in both orientations in the *ara-leu *locus on the chromosome. The BACs used for deletion experiments were generating by inserting fragments of different sizes into the NcoI site of the neo gene. The 4 bp insertion is the same neo* used previously [32]. A 100 bp long cassette having homology to both sides of the insertion was used to restore a functional *neo *gene. The bigger deletion series used a mouse *pax8 *BAC containing a cassette (*neo*) inserted at different distances from a chosen point (0.5, 2, 20, 40, 50 kb). The same *rpsL-bsd *cassette was used for the amplification in the ends-in and ends-out experiment.

### Colony colour test

Part of the *malK *gene (300 bps) was deleted by replacement with the blasticidin resistance gene. This was restored with a cassette containing the missing 300 bp plus 50 bp of further homology to *malK *either side. To obtain a high efficiency of recombination (~1/500 colonies), 1 μg of a PS cassette was electroporated and 100 μl of 10^6 ^dilution were plated on MacConkey agar plus maltose. The experiment was performed at 30°C to minimize the number of replication forks. The restoration of a functional *malK *gave higher efficiency of recombination (probably due to the shorter length of the transformed cassette (400 bp) and was used for the experiment in Table1.

## Authors' contributions

MM, AE, JF and AF performed the experiments. YZ made the initial discovery. MM, AE, JF, YZ and AFS designed the experiments. AFS wrote the paper. All authors approved the final version.

## Supplementary Material

Additional file 1 **Supplementary information**. Oligonucleotides used Click here for file
