# Supplementary material for: Single-stranded heteroduplex intermediates in λ Red homologous recombination
Source: BMC Mol Biol. 2010 Jul 29;11:54. doi: 10.1186/1471-2199-11-54 (PMC2918612; doi:10.1186/1471-2199-11-54)
Supplement: Additional file 1 — Supplementary information. Oligonucleotides used [file 1471-2199-11-54-S1.DOC]

**Supplementary information: Oligonucleotides used**

# Exchange of replication origin

Bsd colE1 up

ccagcaacgcggcctttttacggttcctggccttttgctggAGCACGTGTTGACAATTAATC

Bsd colE1 low gaatcaggggataacgcaggaaagaacatgtgagcaaaaggCTGCTCCTCGGCCACGAAGTGC

R6K rec up AAACTACCGCATTAAAGCTTATCGATGATAAGCTGTCAAACATGAGAATTatttaaaaggatctaggtgaag

R6K rec low GGTCTGAGCTCGCCATCAGTTCAACCTGTTGATAGTACGTACTAAGCTCTCaatcaggggataacgcaggaaa

**Restoration of a functional pir gene**

PirepairPCR-a

TATGGCGCTTGCTCCCATTGATAG

PirepairPCR-b

TTGCCGTGCTTTCTGACTTTCC

# Beta recombination and dsDNA recombination

Casup

GACAATAACCCTGATAAATGCTTCAATAATATTGAAAAAGGAAGAGTATGGCCAAGCCTTTGTCTCAAG

Cas low

AGGGCGCGTAAATCAATCTAAAGTATATATGAGTAAACTTGGTCTGACAGTTAGCCCTCCCACACATAACC

**Single Strand oligo repair (ssOR)**

Neo Ld

GCCAGGCTCAAGGCGCGCATGCCCGACGGCGAGGATCTCGTCGTGACCCATGGCGATGCCTGCTTGCCGAATATCATGGTGGAAAATGGCCGCTTTTCTG

Neo Lg

CAGAAAAGCGGCCATTTTCCACCATGATATTCGGCAAGCAGGCATCGCCATGGGTCACGACGAGATCCTCGCCGTCGGGCATGCGCGCCTTGAGCCTGGC

## Heteroduplex formation

Del-malK-up

ataaaaaagccagggggtggaggatttaagccatctcctgCCATTTTCGGATCTGATCAG

Del-malk-low

AATGACATGTTTTCTGCTACTGACAGGTGGGGATAGAGCGCGCTCCTCGGCCACGAAGTGC

# Importance of 5’ end and mismatches in homology arms

Km 50up gTTCGCCAGGCTCAAGGCGCGCATGCCCGACGGCGAGGATCTCGTCGTGACgaGCTAGTTATTGCTCAGCGG

Km 50low

gATGAATCCAGAAAAGCGGCCATTTTCCACCATGATATTCGGCAAGCAGGCgagTAGAAGGCACAGTCGAGG

Km 30 up

gTTCGCCAGGCTCAAGGCGCGCATGCCCGA

Km 30 low

gATGAATCCAGAAAAGCGGCCATTTTCCAC

# Insert size and recombination

P8 amp up

CAGTCTGTGAGCGACTCCCCGGCGATGCCTCACAACTCGATCAGTGAGTAGGACAAATCCGCCGGG

P8 amp low

CGAGAGGGCTCGGGGGTCCTGGCCCCTCCGCGGTCCTTACCGGATTAAAGTATATATGAGTAAACTTG

Puro 0.5kb up

TGAGACAATAACCCTGATAAATGCTTCAATAATATTGAAAAAGGAAGAGTATGACCGAGTACAAGCCCACGG

Puro 0.5 kb low

GATGATTTCCTCCTTCAGAAGAACTCGTCAAGAAGGCGATAGAAGGCGATTCAGGCACCGGGCTTGCGGG

Km 2kb up

TTAAGCATTGGTAACTGTCAGACCAAGTTTACTCATATATACTTTAGACAGCAAGCGAACCGGAATTG

Km 2kb low

AAATAGAGATCTCAGCCAAGCGGCGCGCGGGATCCGACCCTGATGATTTCCTCCTTCAGAAG

Km 20kb up

CCCTCAGGCAGTTTTGTAGCAGGAATCTGCCGGCCTCTGGACAGCAAGCGAACCGGAATTG

Km 20kb low

GTATGCTAGAGGTCTTTCTTCTGGCTCCTCCCCCTGAGCTTCGATGATTTCCTCCTTCAGAAG

Km 40kb up

CATTCACCCTTCGCCATAAAGCAGGAAACCCCAGAGCTCTCCAGTTGACAGCAAGCGAACCGGAATTG

Km 40kb low

GGAGGGCCTGGCCCGTGAACTGCCCATACACGGAGGCAGCATGGGGATGATTTCCTCCTTCAGAAG

Km 50kb up

GCTCCAACAGGAAGTGAATATTCTGGCAATGCCTACAGCCACACCCGACAGCAAGCGAACCGGAATTG

Km 50kb low

AGGCTGGAGTTGGGGAAGCGCCAGGCCTCACTGTAGGAGGAATACGGATGATTTCCTCCTTCAGAAGAAC

Del AK bsd up

TGAGACAATAACCCTGATAAATGCTTCAATAATATTGAAAAAGGAAGAGTATGGCCAAGCCTTTGTCTCAAG

Del AK bsd low

GATGATTTCCTCCTTCAGAAGAACTCGTCAAGAAGGCGATAGAAGGCGATTTAGCCCTCCCACACATAACCAG

Neo length up

AGGGCGCGTAAATCAATCTAAAGTATATATGAGTAAACTTGGTCTGACAGgcaggtagcttgcagtgggctta

Neo 1kb low

TGAGACAATAACCCTGATAAATGCTTCAATAATATTGAAAAAGGAAGAGTtcagaagaactcgtcaagaagg

Neo 2kb low

TGAGACAATAACCCTGATAAATGCTTCAATAATATTGAAAAAGGAAGAGTttcctcctacatagttggcag

Neo 3kb low

TGAGACAATAACCCTGATAAATGCTTCAATAATATTGAAAAAGGAAGAGTcggaattcctctagagtccag

# Ends out and Ends in recombination

Ends out up

TGTTCGCCAGGCTCAAGGCGCGCATGCCCGACGGCGAGGATCTCGTCGTGACgtaatattctctagacatcattaattcc

Ends out low

TCCAGAAAAGCGGCCATTTTCCACCATGATATTCGGCAAGCAGGCATCGCtagaaggcacagtcgaggtag

Ends in up

GTCACGACGAGATCCTCGCCGTCGGGCATGCGCGCCTTGAGCCTGGCGAACAgtaatattctctagacatcattaattcc

Ends in low

GCGATGCCTGCTTGCCGAATATCATGGTGGAAAATGGCCGCTTTTCTGGAtagaaggcacagtcgaggtag
